# A longitudinal study of adolescent pathways differentiating suicide ideation and attempt in early adulthood

**DOI:** 10.1002/jad.12427

**Published:** 2024-10-21

**Authors:** Geneviève Morneau‐Vaillancourt, Massimiliano Orri, Isabelle Ouellet‐Morin, Marie‐Claude Geoffroy, Michel Boivin

**Affiliations:** ^1^ Social, Genetic & Developmental Psychiatry (SGDP) Centre, Institute of Psychiatry, Psychology & Neuroscience (IoPPN) King's College London London UK; ^2^ School of Criminology, Faculty of Arts and Sciences University of Montreal Montreal Quebec Canada; ^3^ Research Center of the Montreal Mental Health University Institute University of Montreal Montreal Quebec Canada; ^4^ Department of Psychiatry, McGill Group for Suicide Studies, Douglas Mental Health University Institute McGill University Montreal Quebec Canada; ^5^ Department of Epidemiology, Biostatistics, and Occupational Health, School of Population and Global Health McGill University Montreal Quebec Canada; ^6^ Danish Research Institute for Suicide Prevention Copenhagen Mental Health Centre Copenhagen Denmark; ^7^ Department of Educational and Counselling Psychology McGill University Montreal Quebec Canada; ^8^ School of Psychology, Faculty of Social Sciences Laval University Quebec City Quebec Canada

**Keywords:** depressive symptoms, peer victimization, school difficulties, suicidal ideation, suicide attempt

## Abstract

**Objective:**

Suicide ideation and attempt are leading risk factors for mortality in young adults. However, the adolescent risk factors distinguishing suicide ideation from attempt in young adults remain unclear. The present study aimed to examine the extent to which within‐person stability and change in depressive symptoms, school difficulties, and peer victimization from ages 12 to 17 were differentially associated with later suicide ideation and attempt from ages 20 to 23.

**Method:**

The study included 1647 participants from the Quebec Longitudinal Study of Child Development (QLSCD; 52% female). Participants reported on their depressive symptoms, school difficulties, and peer victimization at ages 12, 13, 15, and 17, and on suicide ideation and attempt at ages 20 and 23. Data were collected in the Province of Quebec, Canada, between 2010 and 2021.

**Results:**

Results indicated that 11% (*N* = 121) and 8% (*N* = 86) reported suicide ideation and attempt, respectively, between ages 20 and 23. A random‐intercept cross‐lagged panel model showed that within‐person increases in depressive symptoms during adolescence were related to both suicide ideation and attempt, whereas within‐person increases in school difficulties and peer victimization were for the most part related to suicide attempt only. Within‐person stability in depressive symptoms from ages 12 to 17 years were also related to suicide attempt, and not ideation. However, this association was only marginally significant.

**Conclusion:**

Findings suggest that experiencing unusual rises in school difficulties and peer victimization during adolescence, as well as depressive symptoms persisting over time, may distinguish young adults who think about suicide from those who attempt suicide.

## 1 INTRODUCTION

Youth suicide thoughts and behaviors, including serious suicide ideation and attempts, represent a major public health issue that profoundly affects the lives of young individuals, their family, and friends (Nock et al., 2013; World Health Organization, 2021). Suicide ideation and attempt tend to peak during adolescence and early adulthood and are critical risk factors for suicide mortality, a leading cause of death among individuals aged 15–29 (Bould et al., 2019; Fazel & Runeson, 2020; Nock et al., 2013; World Health Organization, 2021). Approximately 8%–17% of individuals between 14 and 25 years report serious suicide thoughts, while 2%–7% have reported a suicide attempt, with these rates on the rise (Van Meter et al., 2023; Nock et al., 2013; Orri, Scardera, et al., 2020). Therefore, it is essential to identify the early risk factors of suicide in young adults to enhance prevention efforts.

Adolescence is a critical developmental period characterized by significant changes across emotional, academic, and interpersonal domains. Extensive research indicates that difficulties in these areas can substantially increase the risk of suicide ideation and attempts among youth (Castellví et al., 2020; van Geel et al., 2014; Marraccini & Brier, 2017; Miranda‐Mendizabal et al., 2019; Moore et al., 2017; Pozuelo et al., 2022; Soto‐Sanz et al., 2019). Up to 25% of adolescents experience clinically relevant depressive symptoms at some point, making this a sensitive period for the development of depression (Lu, 2019). Population‐based studies indicate that depressive symptoms are associated with suicide ideation and attempt throughout adolescence (Commisso et al., 2023; Lawson et al., 2022; Orri, Scardera, et al., 2020). School is also a central component of adolescents' lives, and large community studies involving thousands of participants indicate that lower academic performance is associated with higher risk of attempting suicide during adolescence (Orozco et al., 2018; Sörberg Wallin et al., 2018). Finally, adolescence is characterized by increased pressures to be socially accepted by peers, and for many, to be popular (Brown & Larson, 2009). Longitudinal population‐based studies show that peer victimization in adolescence increase risk for suicide ideation and attempt (Geoffroy et al., 2016; Perret et al., 2020). These findings are supported by recent meta‐analyses showing that depressive symptoms, school difficulties, and peer victimization are key risk factors for suicide ideation and attempt in young people (Castellví et al., 2020; van Geel et al., 2014; Marraccini & Brier, 2017; Miranda‐Mendizabal et al., 2019; Moore et al., 2017; Pozuelo et al., 2022; Soto‐Sanz et al., 2019). Given the interrelated nature of these difficulties, it is crucial to examine their development and putative impact simultaneously. This multidimensional perspective provides a more comprehensive understanding of the complex pathways that can lead vulnerable youth to suicide behavior in early adulthood.

To effectively prevent youth suicide, it is also crucial to identify the factors that differentiate young people who have suicide thoughts from those who act on those thoughts (Klonsky & May, 2014; Nock et al., 2016). Research indicates that approximately one‐third of young adults who contemplate suicide will eventually attempt it (Nock et al., 2013). Various theories have been proposed to explain the difference between suicide ideation and attempt. Joiner's Interpersonal Theory suggests that feelings of thwarted belongingness and perceived burdensomeness contribute to suicide ideation, while the progression to an attempt is driven by “capability for suicide,” that is an individual's acquired ability to attempt suicide (Van Orden et al., 2010). Building on this model, O'Connor's proposes an Integrated Motivational‐Volitional Theory, which emphasizes that defeat and entrapment lead to suicide ideation, while “volitional moderators,” such as capability for suicide, access to means, exposure to suicide, and impulsivity, facilitate the transition from ideation to attempt (O'Connor, 2011). Finally, Klonsky and May's Three‐Step Theory posits that a combination of emotional pain and hopelessness contributes to suicide ideation, with connectedness serving as a protective factor against the escalation of suicide ideation. According to this theory, the progression from ideation to attempt is explained by dispositional, acquired, and practical contributors to suicide capacity (Klonsky & May, 2015). These theories are all grounded in the ideation‐to‐action framework, suggesting that the progression from suicide ideation to attempt is caused by different predictors and mechanisms. Studies in adult populations support these theories by showing that many factors, such as mental disorders, hopelessness, gender, marital status, and education, are associated with both suicide ideation and attempt, while a subset of factors, including drug use, experiencing abuse, and being exposed to painful and provocative experiences, are uniquely associated with suicide attempt (Kessler et al., 1999; Klonsky et al., 2017; May & Klonsky, 2016).

Despite the importance of the ideation‐to‐action framework in understanding suicide, longitudinal studies examining this framework during adolescence and early adulthood are rare. Some research supports the ideation‐to‐action framework in young populations by showing that adolescent risk factors, like depression, increase the risk for suicide ideation, while only a subset of factors, including experiencing childhood externalizing problems, having a psychiatric disorder, using drugs, or being exposed to self‐harm in others, predict the transition to suicide attempt (Commisso et al., 2023; Mars et al., 2019a, 2019b; Wetherall et al., 2018). Yet, most existing studies have primarily focused on risk factors during childhood or used a cross‐sectional design, leaving a critical gap in how these factors evolve over time. It remains unclear whether a comprehensive examination of various risk factors throughout adolescence—a crucial period for suicide risk—could provide insights into differentiating long‐term risks for suicide ideation and attempt.

Understanding the timing and extent to which fluctuations in depressive symptoms, academic difficulties, and peer victimization increase the risk for suicide ideation and attempt could help identify sensitive window periods for intervention. To achieve this, it is essential to adopt a within‐person design that focuses on intraindividual patterns of stability and change over time (Curran & Bauer, 2011). Yet, previous longitudinal studies often used between‐person designs examining individual differences over time (i.e., how individuals evolve compared to others) rather than within‐person designs examining how individuals evolve over time compared to themselves. As such, little is known about the role of transient (i.e., within‐person change) versus more persistent (i.e., within‐person stability) depressive symptoms in predicting suicide ideation and attempt. A few longitudinal studies show that within‐person increases in depressive symptoms, basic psychological need satisfaction at school, and peer victimization are prospectively associated with later suicide ideation during adolescence and early adulthood (Chen et al., 2022; Miller et al., 2017; Yang et al., 2024; Ye et al., 2024; Zhu et al. 2022a, 2022b). However, these studies have primarily focused on suicide ideation, with only one study separately measuring both suicide ideation and attempt in a sample of 220 adolescent girls (Miller et al., 2017). Expanding research within the ideation‐to‐action framework to include longitudinal, within‐person analyses could significantly enhance our understanding of the complex interplay of risk factors in youth suicide.

The present study aimed to address this gap by examining the extent to which within‐person stability and change in depressive symptoms, school difficulties, and peer victimization from ages 12 to 17 differentiate young adults who think about suicide from those who attempt suicide between ages 20 and 23. We used longitudinal cross‐lagged analytical approaches to disaggregate within‐ from between‐person longitudinal associations (Curran & Bauer, 2011), and examine the extent to which within‐person stability and change in various forms of adversity across adolescence would be prospectively associated with suicide ideation and attempt in early adulthood. We expected that within‐person increases in depressive symptoms would be related to both suicide ideation and attempt, and that the other two forms of adversity, school difficulties and peer victimization, would be associated with suicide attempt.

## 2 METHODS

### 2.1 Participants

We used data from the Quebec Longitudinal Study of Child Development (QLSCD), an ongoing population‐based cohort study conducted by the *Institut de la Statistique du Québec* (Orri, Boivin, et al., 2020). Initially, 2120 families with a child born between 1997 and 1998 in the Province of Quebec, Canada, were recruited. Participants and their families were followed almost annually over more than 25 years. Ethics committees of the *Institut de la Statistique du Québec* approved each phase of the study. The 2021 Special Round data collection (23 years) wave was also approved by the Douglas Research Centre and CHU Ste‐Justine research ethics committees in Montreal. Informed consent was obtained from participants and/or their parents at each data collection. Further details about the cohort can be found online (https://www.jesuisjeserai.stat.gouv.qc.ca).

### 2.2 Missing data

The present study includes 1647 participants who had at least one available measure across ages 13, 15, 17, 20, or 23, out of the 2120 participants initially recruited (78% of original sample). At each assessment wave, the available number of participants varied between 59% and 66% of the initial 2120 participants, as presented in the cohort profile (Orri, Boivin, et al., 2020). Specifically, the number of participants at each assessment wave was 1396 at 12 years old; 1290 at 13 years old; 1399 at 15 years old; 1252 at 17 years old; 1399 at 20 years old; and 1368 at 23 years old. The percentage of available data in the present study, based on the available sample at each assessment wave (*N* = 1252–1399), varied from 88% to 100%, meaning that most participants answered questions related to the measures of interest. Table 1 also present socioeconomic and demographic differences between participants who were included (*N* = 1647) and those who were not (*N* = 2120–1647 = 473). Participants who were included were more likely to be females, to have mothers who were educated beyond high school, to have a minimum of one parent employed when they were born, and to come from French‐speaking families. Participants who were included also had higher socioeconomic status and their mothers had lower depression scores. Surprisingly, participants who were included showed lower school performance in childhood compared to participants who were not included. There were no differences between participants who were included and those who were not regarding verbal IQ at age 5 years, separation anxiety in early childhood, age of mother and father at birth, fathers' education level, having an intact family at birth, and father's depression. Overall, participants who were included in the present study were more likely to be females from French‐speaking and higher socioeconomic backgrounds and less likely to have a mother experiencing depression, compared to the initial QLSCD sample.

**Table 1:** Sociodemographic characteristics of the QLSCD and descriptive statistics of main variables.^a^

	Participants included (total *N* = 1647)^b^	Participants not included (total *N* = 473)	*t* test	*χ* ^2^
Participant characteristics				
Females (%)	0.52 (*N* = 852)	0.40 (*N* = 188)	‐	20.64*
Verbal IQ at age 5 years,^c^ mean (SD)	115.28 (17.18) (*N* = 1091)	113.61 (17.13) (*N* = 74)	0.81	‐
Separation anxiety in early childhood,^d^ mean (SD)	2.63 (1.49) (*N* = 1647)	2.72 (1.68) (*N* = 410)	−0.94	‐
School performance in childhood,^e^ mean (SD)	2.53 (1.09) (*N* = 1438)	2.97 (1.24) (*N* = 92)	−3.76*	‐
Family characteristics when participants were 5 months old				
Older than age 20 years at child's birth (%)				
Mother	0.97 (*N* = 1647)	0.96 (*N* = 472)	‐	1.14
Father	0.92 (*N* = 1528)	0.90 (*N* = 422)	‐	1.31
Education beyond high school (%)				
Mother	0.85 (*N* = 1646)	0.80 (*N* = 471)	‐	7.34*
Father	0.83 (*N* = 1260)	0.80 (*N* = 333)	‐	1.60
Minimum one parent employed (%)	0.93 (*N* = 1635)	0.87 (*N* = 467)	‐	12.40*
Language spoken at home is French (%)	0.88 (*N* = 1609)	0.76 (*N* = 454)	‐	44.39*
Family is intact at child's birth^f^ (%)	0.82 (*N* = 1644)	0.80 (N = 471)	‐	1.15
Socioeconomic status,^g^ mean (SD)	0.05 (1.00) (*N* = 1633)	−0.22 (0.99) (*N* = 462)	5.20*	‐
Parental depression,^h^ mean (SD)				
Mother	1.37 (1.32) (*N* = 1642)	1.52 (1.42) (*N* = 471)	−2.06*	‐
Father	0.99 (0.94) (*N* = 1413)	1.05 (1.04) (*N* = 357)	−1.16	‐
Main variables included in the study				
Depressive symptoms age 12 (*N* available)	2.43 (2.83) (*N* = 1346)	‐	‐	‐
Depressive symptoms age 13 (*N* available)	2.86 (3.16) (*N* = 1228)	‐	‐	‐
Depressive symptoms age 15 (N available)	5.58 (3.60) (N = 1,442)	‐	‐	‐
Depressive symptoms age 17 (*N* available)	6.10 (3.71) (*N* = 1264)	‐	‐	‐
School difficulties age 12 (*N* available)	0.17 (0.21) (*N* = 1,332)	‐	‐	‐
School difficulties age 13 (*N* available)	0.19 (0.20) (*N* = 1175)	‐	‐	‐
School difficulties age 15 (*N* available)	0.27 (0.27) (*N* = 1433)	‐	‐	‐
School difficulties age 17 (*N* available)	0.25 (0.26) (*N* = 1130)	‐	‐	‐
Peer victimization age 12 (*N* available)	3.13 (2.59) (*N* = 1343)	‐	‐	‐
Peer victimization age 13 (*N* available)	2.43 (2.73) (*N* = 1229)	‐	‐	‐
Peer victimization age 15 (*N* available)	2.20 (2.63) (*N* = 1436)	‐	‐	‐
Peer victimization age 17 (*N* available)	1.71 (2.28) (*N* = 1227)	‐	‐	‐
Suicide ideation age 20, % (*N* available)	0.07 (*N* = 1229)	‐	‐	‐
Suicide ideation age 23, % (*N* available)	0.07 (*N* = 1317)	‐	‐	
Suicide attempt age 20, % (*N* available)	0.06 (*N* = 1229)	‐	‐	‐
Suicide attempt age 23, % (*N* available)	0.02 (*N* = 1317)	‐	‐	‐

Abbreviations: CBCL, Child Behavior Checklist; CES‐D, Center for Epidemiological Studies Depressive Scale; *N*, number of participants; QLSCD, Quebec Longitudinal Study of Child Development; RI‐CLPM, random‐intercept cross‐lagged panel model; SD, standard deviation.

^a^ Data were compiled from the final master file of the QLSCD.

^b^ Number of participants included in the final RI‐CLPM. Participants with at least one available assessment of depressive symptoms, school difficulties, peer victimization, suicide ideation, or suicide attempt were included as the model was estimated using missing = “pairwise” and estimator = “WLSMV” in lavaan.

^c^ Verbal IQ was assessed using a French adaptation of the Peabody Picture Vocabulary Test (Dunn et al., 1993), range from 0 to 160 with a higher score representing higher verbal IQ. The high verbal IQ scores in our sample (approximately 115 vs. a typical mean of 100) could be explained by the fact that the norms for this test were developed using scores from French‐speaking children in Canada. Children in the QLSCD may show higher levels of French, as most of them lived in a French‐speaking environment, while French‐speaking children from other parts of Canada are more likely to live in an English‐speaking environment (except for children from other French areas outside Quebec).

^d^ Separation anxiety was assessed using parent reports at ages 1.5, 2.5, 3.5, 4, 5, and 6 years. At each time point, three items adapted from the Child Behavior Checklist (CBCL/11/2‐5; Achenbach, 1991) were rated on a 3‐point scale, with 0 = “never or rarely”; 1 = “sometimes”; 2 = “often.” Items were “Does your child… (1) react badly when a parent is away; (2) not want to sleep alone; (3) cling to adults and is too dependent.” A composite score was aggregated by computing an average across ages 1.5, 2.5, 3.5, 4, 5, and 6 years. Higher scores represented higher separation anxiety.

^e^ School performance was assessed using teacher reports at ages 7, 8, and 10 years. Teachers answered the following question: “How would you rate this child's current academic achievement across all areas of instruction?” on a five‐point scale, with 1 = “Near the top of the class”; 2 = “Above the middle of the class, but not at the top”; 3 = “In the middle of the class”; 4 = “Below the middle of the class, but above the bottom”; 5 = “Near the bottom of the class.” Higher scores represented lower school performance.

^f^ An intact family was defined as both biological parents being together at the child's birth.

^g^ Socioeconomic status was assessed using a composite score of five items measuring parental educational level, parental occupation, and annual gross income with higher scores representing higher socioeconomic status.

^h^ Parental depressive symptoms were assessed using the short 12‐item version of the CES‐D (Radloff, 1977), range from 0 to 10 with higher scores representing more depressive symptoms.

* *p* < .01.

### 2.3 Measures

#### 2.3.1 Depressive symptoms at ages 12, 13, 15, and 17

Participants answered 10 questions from the Child Depressive Inventory short‐form (CDI‐SF) (Kovacs, 1992), plus four additional items, at ages 12 and 13. At ages 15 and 17, they answered eight questions from the Mental Health and Social Inadaptation Assessment for Adolescents (MIA) (Côté et al., 2017). Items from the CDI‐SF and MIA are comparable, both answered on a three‐point scale. Both scales have been shown reliable and valid (Carey et al., 1987; Côté et al., 2017; Timbremont et al., 2004). The CIDI‐SF asked how participants felt during the past 2 weeks, for example: “I'm sad sometimes; I'm sad often; I'm sad all the time.” The MIA asked participants the extent to which they experienced depressive symptoms during the past 12 months, for example “I felt sad and unhappy.” Scores were computed by averaging the items. Cronbach's *⍺* coefficients were .87, .78, .87, and .88 at ages 12, 13, 15, and 17, respectively.

#### 2.3.2 School difficulties at ages 12, 13, 15, and 17

School difficulties were assessed using the Dropout Prediction Index (Archambault & Janosz, 2009), a multidimensional self‐assessment scale based on work by Janosz et al. (1997, 2000). The Dropout Prediction Index has been validated and used previously (Archambault & Janosz, 2009; Fitzpatrick et al., 2015). It includes three dimensions: two items assessing school performance in French/English Language Arts and mathematics (0–100), one item assessing how many times participants had failed a school year (0, 1, 2, 3 or more times), and four items from the validated *Mesures de l'adaptation sociale et personnelle pour les adolescents québécois* (LeBlanc, 1998) assessing school engagement. The three subscales were weighted based on previous work and then added to create a single score, which was converted on a 0‐to‐1 probability scale for dropping out (see Supporting Information).

#### 2.3.3 Peer victimization at ages 12, 13, 15, and 17

Peer victimization was assessed using an adapted version of the Self‐Report Victimization Scale (Kochenderfer & Ladd, 1996). The scale has good convergent validity with related constructs such as loneliness and peer rejection (Ladd & Kochenderfer‐Ladd, 2002). Participants had to answer six questions about how many times a situation happened to them at school, such as “Someone called me names, insulted me or said mean things to me.” At age 12, items were rated on a three‐point scale: 0 for “never,” 1 for “one or two times,” and 2 for “more often.” At ages 13, 15, and 17, items were rated on a four‐point scale: 0 for “never,” 1 for “rarely (one or two times),” 2 for “often (one time per week),” and 3 for “very often (more than one time per week).” At each time point, an average score across items was calculated, and scores were converted to a 0‐to‐10 scale. Cronbach's *⍺* coefficients were .86, .81, .88, and .96 at ages 12, 13, 15, and 17, respectively.

#### 2.3.4 Suicide ideation and suicide attempt

Suicide ideation and attempt were assessed at ages 20 and 23. Participants were asked whether, in the past 12 months, they “seriously thought of attempting suicide” and if so, whether they “attempted suicide” (at age 20) or “how many times they attempted suicide” (at age 23). Participants were coded 1 for suicide ideation if they answered “yes” to the first question about suicide thoughts and “no” to the questions about suicide attempt (suicide ideation without attempt). Participants were coded 1 for suicide attempt if they answered “yes” or “at least once” to the questions about suicide attempt. As in previous studies (Forte et al., 2021; Galera et al., 2021; Orri, Boivin, et al., 2020; Orri, Scardera, et al., 2020; Orri et al., 2021, 2018, 2021, 2018), an overall suicide ideation variable across ages 20 and 23 was computed, with 1 = at least one report of suicide ideation (without attempt) and 0 = never reported suicide ideation. The same was done for suicide attempt.

At ages 13, 15, and 17, the same questions about suicide ideation and attempt were available. We used these variables to run sensitivity analyses to control for previous suicide ideation and attempt (analyses are further described below). We computed two new variables, one for suicide ideation and one for suicide attempt. We identified participants who had not reported suicide ideation or attempt between ages 13 and 17, but who did report suicide ideation or attempt for the first time at ages 20 or 23 (coded 1). We also identified participants who never reported suicide ideation or attempt between ages 13 and 23 (coded 0). Participants who had at least one available assessment at ages 13, 15, or 17 as well as at ages 20 or 23 were included. Descriptive statistics and correlations between suicide variables are presented in Supporting Information S1: Table S2.

### 2.4 Data analyses

We performed descriptive analyses using R (version 4.2.3) (R Core Team, 2017) and structural equation models using lavaan (Rosseel, 2012). Structural equation models were estimated using options missing = “pairwise” and estimator = “WLSMV” to account for missing data and the fact that the two outcomes, suicide ideation and attempt, were binary variables. Paths predicting suicide ideation and attempt were estimated using a probit model, assuming that a normally distributed latent response variable was underlying the binary variables. We then converted these estimates to probabilities using a formula provided by Muthén and Muthén (Muthén & Muthén, 2009) to facilitate interpretation of probit coefficients (further explained in the Supporting Information). We assessed model fit using the Root Mean Square Error of Approximation (RMSEA), Comparative Fit Index (CFI), and Tucker–Lewis Index (TLI).

We first ran a cross‐lagged panel model (CLPM) to examine the bidirectional associations between depressive symptoms, school difficulties, and peer victimization over time, and included suicide ideation and attempt as outcomes. The CLPM documents the reciprocal predictive associations between variables by controlling for their stability over time and pre‐existing correlations. However, the CLPM is limited in that it cannot distinguish between‐ from within‐person longitudinal associations (Hamaker et al., 2015). To overcome this limitation, we conducted a random‐intercept cross‐lagged panel model (RI‐CLPM). For each variable measured repeatedly over time, the RI‐CLPM estimates a latent intercept factor that represents stable between‐person variance accounting for unobserved influences from stable and trait‐like factors. As such, the RI‐CLPM accounts for any stable socioeconomic characteristic that may play a role in predicting suicide outcomes, such as belonging to a minority group and exposure to self‐harm in others in the past (Klonsky et al., 2016). By doing so, the model allows examining within‐person fluctuations over time, compared to individuals' baseline or average level, as well as how these within‐person fluctuations may impact later suicide ideation and attempt (Hamaker et al., 2015; Mulder & Hamaker, 2021). By using a longitudinal design covering adolescence to emerging adulthood, we were able to examine sensitive periods during which within‐person fluctuations in key risk factor may increase risk for suicide ideation and attempt.

## 3 RESULTS

The study included 1647 participants who had a minimum of one available time point (using missing = “pairwise” and estimator = “WLSMV” in lavaan). Table 1 presents sociodemographic characteristics for QLSCD participants who were included in the present study versus those who were not, as well as descriptive statistics for the main variables. Rates of suicide ideation were 7% (*N* = 84) at age 20 and 7% (*N* = 93) at age 23. Rates of suicide attempt were 6% (*N* = 72) at age 20 and 2% (*N* = 22) at age 23. The overall rates for reporting suicide ideation and attempt at least once across the two waves were 11% (*N* = 121) and 8% (*N* = 86), respectively. Table 2 presents Pearson and point‐biserial correlations between variables.

**Table 2:** Correlations between variables in QLSCD.^a^

	1.	2.	3.	4.	5.	6.	7.	8.	9.	10.	11.	12.
1. Depressive symptoms 12	1											
2. Depressive symptoms 13	0.54*	1										
3. Depressive symptoms 15	0.32*	0.44*	1									
4. Depressive symptoms 17	0.31*	0.39*	0.59*	1								
5. School difficulties 12	0.23*	0.14*	−0.01	0.02	1							
6. School difficulties 13	0.21*	0.26*	0.06	0.06	0.63*	1						
7. School difficulties 15	0.19*	0.16*	0.08*	0.08*	0.62*	0.67*	1					
8. School difficulties 17	0.15*	0.11*	0.01	0.07*	0.55*	0.61*	0.74*	1				
9. Peer victimization 12	0.36*	0.28*	0.22*	0.20*	0.13*	0.13*	0.15*	0.16*	1			
10. Peer victimization 13	0.34*	0.42*	0.24*	0.19*	0.15*	0.21*	0.18*	0.20*	0.51*	1		
11. Peer victimization 15	0.21*	0.23*	0.33*	0.23*	0.07*	0.04	0.09*	0.08*	0.39*	0.45*	1	
12. Peer victimization 17	0.16*	0.17*	0.19*	0.30*	0.08*	0.04	0.11*	0.10*	0.37*	0.34*	0.46*	1
13. Suicide ideation 20–23	0.15*	0.11*	0.11*	0.15*	0.03	−0.00	0.05	0.02	0.06	0.04	0.02	0.08*
14. Suicide attempt 20–23	0.15*	0.21*	0.19*	0.19*	0.15*	0.20*	0.13*	0.13*	0.14*	0.18*	0.15*	0.16*

*Note*: Pearson's correlations were conducted between depressive symptoms, school difficulties, and peer victimization. Point‐biserial correlations were conducted between depressive symptoms, school difficulties, peer victimization and suicide ideation and suicide attempt.

Abbreviation: QLSCD, Quebec Longitudinal Study of Child Development.

^a^ Data were compiled from the final master file of the QLSCD.

* *p* < .05.

**Figure 1:**
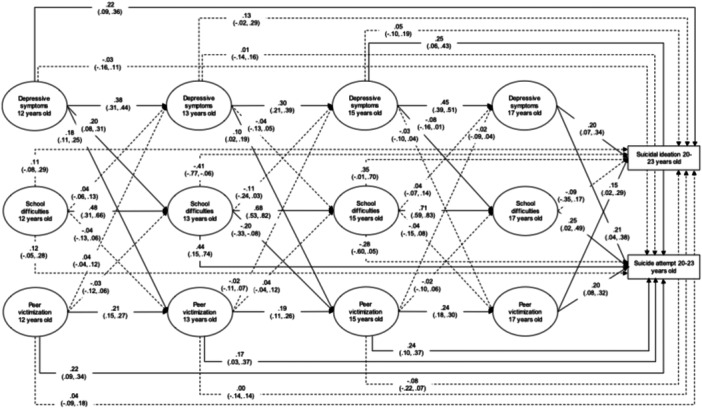
Random‐intercept cross‐lagged panel model predicting suicide ideation and suicide attempt. Full lines represent statistically significant paths, dashed lines, nonsignificant paths. Latent variables represent within‐person components. See Supporting Information S1: Figure S1 for the other estimates not shown here for clarity.

**Figure 2:**
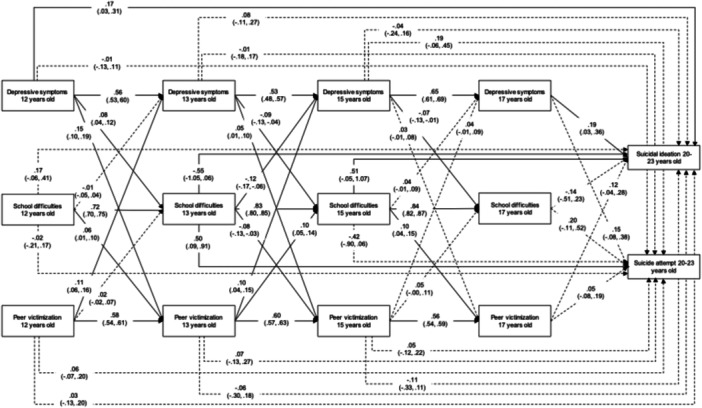
Cross‐lagged panel model predicting suicide ideation and attempt. Full lines represent statistically significant paths, dashed lines, nonsignificant paths. See Supporting Information S1: Figure S2 for the estimates not shown here for clarity.

Both RI‐CLPM (Figure 1) and CLPM (Figure 2) showed adequate fit (RMSEA < 0.04; CFI > 0.99; TLI > 0.97). However, the RI‐CLPM showed a better fit than the CLPM, indicating that including random intercepts and within‐person components improved model fit (*χ*bar^2^ difference = 92.06, degrees of freedom difference = 6, *p* < .001) (Stoel et al., 2006). Thus, accounting for stable, trait‐like factors associated with depressive symptoms, school difficulties, and peer victimization over time improved model fit, with the implication that CLPM results were likely confounded by these unmeasured trait‐like factors. Therefore, we focus on findings from the RI‐CLPM.

The RI‐CLPM showed 11 significant direct effects from adolescent risk factors to suicide ideation or suicide attempt (Figure 1).

### 3.1 Associations with suicide ideation

First, we found that a within‐person increases in depressive symptoms at ages 12 and 17 were associated with a higher probability of reporting later suicide ideation. Given the controls provided by the RI‐CLPM, these associations indicate that higher‐than‐usual depressive symptoms (compared to individuals' average depressive symptoms across time) predicted a higher probability of suicide ideation and attempt. When transforming probit coefficients to probabilities, the probability of reporting suicide ideation increased from 8% (at baseline level) to 12% if participants' depressive symptoms at age 12 were one standard deviation above their overall mean in depressive symptoms across time points. For depressive symptoms at age 17, this probability increased from 8% to 11%. Second, higher‐than‐usual peer victimization at age 17 was associated with suicide ideation, with the probability of reporting suicide ideation increasing from 8% (at baseline level) to 10% if participants scored one standard deviation above their overall mean. School difficulties were not significantly associated with suicide ideation.

### 3.2 Associations with suicide attempt

By comparison, within‐person increases in depressive symptoms later in adolescence, at ages 15 and 17, were associated with suicide attempt. The probability of reporting a suicide attempt increased from 1% (at baseline level) to 2% if participants' depressive symptoms at ages 15 or 17 were one standard deviation above their overall mean in depressive symptoms across time points. We also found that higher‐than‐usual school difficulties at age 13 and 17 were associated with a higher probability of suicide attempt. If participants'school difficulties at age 13 were one standard deviation above their overall mean, their probability of reporting a suicide attempt increased from 1% (at baseline level) to 5%. At age 17, this probability increased from 1% to 2%. Finally, higher‐than‐usual peer victimization at ages 12, 13, 15, and 17 increased the probability of reporting suicide attempt, from 1% (at baseline level) to 2% for a one standard deviation increase from participants' own mean across time points.

We examined whether indirect pathways involving auto‐regressive or cross‐lagged paths and starting at age 12 predicted suicide ideation and attempt. We tested indirect effects based on significant paths in the RI‐CLPM, for a total of nine potential indirect effects (see Supporting Information S1: Table S1). We estimated indirect effects and their 95% confidence intervals (CIs) by running 1000 bootstrapping draws using the options se = “bootstrap” and test = “scaled.shifted” in lavaan. Only one indirect effect was significant according to the bootstrapped CIs: depressive symptoms at age 12 were associated with later suicide attempt via within‐person stability in depressive symptoms from ages 12 to 17 (*Β* = 0.01; 95% CI = 0.0001, 0.02; *p* = .048). However, the effect size was small, the 95% CI was close to 0, and the *p* value was only slightly below .05.

### 3.3 Sensitivity analyses

We ran sensitivity analyses to examine (1) whether accounting for previous suicide ideation and attempt assessed at ages 13, 15, and 17 affected the results, (2) whether pathways predicting suicide ideation versus attempt significantly differed, and 3) whether the patterns of associations were similar across sexes.

We found that 68 participants who had never reported suicide ideation between ages 13 and 17 did report suicide ideation for the first time between ages 20 or 23 (coded 1). A total of 665 participants never reported suicide ideation at ages 13, 15, 17, 20, and 23 (coded 0). For suicide attempt, 29 participants reported attempting suicide for the first time between ages 20 and 23 (coded 1), and 692 participants never reported suicide attempt between ages 13 and 23 (coded 0). We ran the RI‐CLPM again using these variables to examine whether considering previous suicide ideation and attempt at ages 13, 15, and 17 affected the results. Findings are presented in Supporting Information S1: Figure S3A,B and were similar for the most part, suggesting that controlling for previous suicide ideation and attempt did not affect the main results. Predictive associations between depressive symptoms, school difficulties, peer victimization and suicide ideation and attempt at ages 20–23 remained similar for the most part. Five associations with suicide attempt were no longer significant; however, effect sizes did not change considerably. For example, the path from depressive symptoms at age 15 to suicide attempt at ages 20–23 was 0.25 (*p* < .05) in the main model, and now became 0.15 (*p* > .05) after controlling for previous suicide attempt. A similar pattern was found for depressive symptoms at age 17 (0.21, *p* < .05 previously, now 0.19 *p* > .05), school difficulties at age 13 (0.44, *p* < .05 previously, now 0.23 *p* > .05), school difficulties at age 17 (0.25, *p* < .05 previously, now 0.21 *p* > .05), and peer victimization at age 13 (0.17, *p* < .05 previously, now 0.15 *p* > .05). These analyses controlling for previous suicide ideation and attempt were conducted with a smaller sample size. Therefore, the fact that some associations with suicide attempt were no longer significant may be due to lower statistical power, especially when considering that rates of suicide attempts were low (4%).

Finally, we examined whether paths predicting suicide ideation and attempt could be constrained to equality without affecting model fit. We found that a model in which corresponding paths to suicide ideation versus suicide attempt were constrained to equality showed poorer fit than a model in which paths were freely estimated, suggesting that suicide ideation and attempt have different developmental pathways (*χ* ^2^ (12) = 39.86, *p* < .001). We also examined whether constraining paths predicting suicide ideation and attempt to equality across sexes affected model fit. We found no significant difference between models, suggesting no sex differences (*χ* ^2^ (24) = 25.21, *p* = .39).

## 4 DISCUSSION

This longitudinal population‐based study examined the extent to which adolescent depressive symptoms, school difficulties, and peer victimization differentiated the risk for suicide ideation and attempt in early adulthood. This study extends current knowledge of the ideation‐to‐action framework by providing repeated follow‐ups over more than 10 years during adolescence and early adulthood (12–23 years old) and using a random‐intercept cross‐lagged approach examining associations at the within‐person level. Although findings indicate that a few pathways were common to suicide ideation and attempt, suicide ideation and attempt mostly had different developmental pathways. Findings contribute to current knowledge in three ways.

First, the study sheds light on the developmental pathways that differentiate young adults who contemplate suicide from those who act on those thoughts. Higher‐than‐usual school difficulties and peer victimization were associated with later suicide attempt, but not ideation (except peer victimization at age 17, which was also related to suicide ideation). Specifically, within‐person increases in school difficulties at 13 and 17 years old and in peer victimization at 12, 13, 15, and 17 years old were related to later suicide attempt, measured at 20 or 23 years old. This indicates that adolescents who experienced higher‐than‐usual school difficulties or peer victimization at these ages, compared to their own average, were more likely to report having attempted suicide between ages 20 and 23. This finding is consistent with existing studies showing that school difficulties and peer victimization may differentiate adolescent suicide ideation and attempt (Taliaferro & Muehlenkamp, 2014; Vergara et al., 2019). It also adds to current knowledge by suggesting that both academic and interpersonal difficulties uniquely contributed to suicide attempt, despite both types of adversity being correlated. This stresses the importance of considering both risk factors separately when monitoring risk for suicide.

Results also support theories grounded in the ideation‐to‐action framework. School difficulties and peer victimization may act as volitional moderators that may influence decision‐making process regarding suicide, as suggested by the Integrated Motivational‐Volitional Theory (O'Connor, 2011). Research shows that both school difficulties and peer victimization significantly contribute to adolescent psychological distress and social isolation (Almeida et al., 2021; Buhs et al., 2006), which in turn may increase vulnerability by limiting access to supportive relationships with peers and adults and leading to a sense of failure and hopelessness (Lay‐Yee et al., 2023). In addition, by their interpersonal dimension, these factors can contribute to feelings of thwarted belongingness and perceived burdensomeness, consistent with Joiner's Interpersonal Theory (Van Orden et al., 2010). School difficulties and peer victimization may also indicate deeper, underlying problems that translate into severe sources of distress for adolescents. Existing research has identified several factors—such as impulsivity, substance use, exposure to suicide behaviors or self‐harm in peers, and traumatic experiences like sexual abuse—that are associated with increased risk of suicide attempt among young people (Dhingra et al., 2015; Mars et al., 2019a; Wetherall et al., 2018). As these factors often intersect with school difficulties and peer victimization (Hébert et al., 2016; Reijntjes et al., 2011), the latter may serve as observable markers of other significant issues. Importantly, school difficulties and peer victimization are readily identifiable within the education environment, suggesting their potential utility as initial screening indicators for suicide risk.

Further supporting the ideation‐to‐action framework is the finding that stability in depressive symptoms from ages 12 to 17 years was associated specifically with suicide attempt, rather than suicide ideation. Adolescents who consistently exhibit high levels of depressive symptoms may become habituated to psychological pain due to their ongoing mental health struggles. This habituation could lead to an acquired capability to attempt suicide, differentiating between suicide ideation and actual attempt, as proposed by the Integrated Motivational‐Volitional Theory and Three‐Step Theory (Klonsky & May, 2015; O'Connor, 2011). This finding must however be interpreted with caution, as the effect size was small and slightly below the threshold for statistical significance, indicating a need for replication in future research. Nonetheless, it aligns with previous evidence showing that a trajectory of persistent and comorbid internalizing and externalizing problems during childhood differentiated suicide attempt from ideation at 22 years old (Commisso et al., 2023). Results suggest that monitoring adolescents with persistent elevated depressive symptoms may be an effective strategy for identifying those at risk for suicide attempt.

Second, the study enhances our understanding of adolescent risk factors for suicide by using a within‐person approach, offering a detailed examination of intraindividual patterns of stability and change over time. A critical question that remained unanswered was whether fluctuations across emotional, academic, and interpersonal over time could predict higher risks of suicide ideation and attempt. Our findings revealed significant associations, indicating that time‐specific increases in difficulties across these domains were related to suicide ideation and attempt. They also showed that, in addition to fluctuations, the stability in depressive symptoms over the years uniquely contributed to suicide attempt. This underscores the importance of monitoring unusual rises but also stable patterns of adolescent difficulties when assessing risk. Focusing on how difficulties evolve—whether they change or persist—may provide a better understanding of an individual's risk for suicide ideation and attempt.

Finally, the study significantly contributes to our understanding of adolescent risk factors for suicide by providing an extended longitudinal follow‐up that tracks how these factors evolve throughout adolescence. Rather than identifying a single sensitive period, the findings indicate that risk factors present at various time points during adolescence contribute to later suicide ideation and attempt. For example, within‐person increase in peer victimization at ages 12, 13, 15, and 17 were related to suicide attempt. This suggests that continuously monitoring these risk factors at any stage during adolescence is essential to identify youths who may be at increased risk of suicide ideation and attempt.

## 5 LIMITATIONS

The present study has four main limitations. First, all measures were self‐reported and rater bias may have inflated the associations through shared common variance. Second, despite being correlated (*r* = .31–.44) and including comparable items, different scales were used to assess depressive symptoms at ages 12 and 13 (CDI‐SF) and ages 15 and 17 (MIA). The assessment of depressive symptoms at ages 12 and 13 may have been qualitatively different from that at ages 15 and 17, as the retrospective window to evaluate depressive symptoms was different (i.e., past 2 weeks vs. past 12 months). Third, when interpreting results in terms of probabilities, we found that within‐person increases in risk factors (e.g., one standard deviation) only represented small increases in probabilities of reporting suicide ideation and attempt (e.g., from 1% to 2%). This is related to the fact that the proportion of QLSCD participants who reported suicide ideation and attempt was low, as this is a population‐based study. Results must be interpreted while considering these small effect sizes. Fourth, the generalization of findings to minority groups and individuals from different socioeconomic backgrounds is limited. At inception in 1998, QLSCD mostly included White participants and did not include indigenous populations. In addition, more females and participants from higher socioeconomic backgrounds remained involved in QLSCD over the years. This limits generalization of findings to other groups in the population. Nonetheless, the random‐intercept cross‐lagged approach accounted for any source of confounding factors that are stable over time through the inclusion of random intercepts. As such, findings could not be explained by differences in sociodemographic characteristics between participants.

## 6 CONCLUSIONS

The study used a prospective longitudinal design to examine the developmental pathways that differentiate suicide ideation from attempt in a population‐based sample of young adults. Findings showed that different developmental pathways distinguished young adults who thought about suicide from those who attempted suicide. Unusual rises in school difficulties and peer victimization during adolescence, as well as persisting depressive symptoms throughout adolescence, were uniquely associated with suicide attempt, and not ideation. Findings underscore the necessity of monitoring these difficulties, as well as how they evolve over time, to more accurately prevent suicide ideation and attempt.

## CONFLICT OF INTEREST STATEMENT

The authors declare no conflict of interest.

## Supporting information

Supporting information.

## Data Availability

Geneviève Morneau‐Vaillancourt had full access to all the data in the study and takes responsibility for the integrity of the data and the accuracy of the data analysis. The data that support the findings of this study are available on request from the Research Data Access Point team of the *Institut de la statistique du Québec*. More information on how to access the data is provided here https://jesuisjeserai.stat.gouv.qc.ca/informations_chercheurs/acces_an.html. The data are not publicly available due to privacy or ethical restrictions.
