# Supplementary material for: A longitudinal study of adolescent pathways differentiating suicide ideation and attempt in early adulthood
Source: J Adolesc. 2024 Oct 21;97(2):395–408. doi: 10.1002/jad.12427 (PMC11791732; doi:10.1002/jad.12427)
Supplement: Supplementary file 1 — Supporting information. [file JAD-97-395-s001.docx]

**Supplementary Materials**

**Dropout Prediction Index Measure**

The Dropout Prediction Index was computed at ages 12, 13, 15, and 17 and included three subscales: school performance, retention, and school engagement. Items for the school performance subscale were: 1) *During this school year, what is your average mark in French/English Language Arts?* 2) *During this school year, what is your average mark in mathematics?* School retention was evaluated using the following item: 3) *Have you ever repeated an entire school year?* 0 = *never*; 1 = *once*; 2 = *twice*; 3 = *three times or more*. Items for the school engagement subscale were: 4) *Do you like school?* 1 = *I don't like school at all*; 2 = *I don't like school*; 4 = *I like school*; 5 = *I really like school*. 5) *In terms of your school marks, how would you rate yourself compared with other students your age at your school?* 1 = *I am one of the weaker students*; 2 = *I am weaker than the average student*; 3 = *I am an average student*; 4 = *I am stronger than the average student*; 5 = *I am one of the stronger students*. 6) *How important is it for you to get good marks?* 1 = *not important at all*; 2 = *somewhat important*; 3 = *important*; 4 = *very important*. 7) *Based on your own wishes, how far do you plan to go in school?* 1 = *I don't know. It doesn't bother me*; 2 = *I plan to leave before completing high school*; 3 = *I plan to finish high school (general education)*; 3 = *I plan to do vocational training in high school*; 4 = *I plan to do technical training at the CEGEP level (junior college)*; 5 = *I plan to attend university.*

Each subscale was weighted by a coefficient representing their respective prediction of school dropout from logistic regression models reported in previous studies (Janosz et al., 1997, 2000). The following formulas were used to compute the three subscales, in which the constant 8.81 was determined based on the rate of school dropout in the Quebec Longitudinal Study of Child Development.

$$school performance = ([item1 + item2]\div2) \times-.095$$

$$school retention = item3 \times1.13$$

$$school engagement = (\Sigma item4 + item5 + item6 + item7) \times-.246$$

$$total= school performance + school retention + school engagement + 8.81$$

$$dropout risk score = \frac{e^{total}}{1 + e^{total}}$$

We examined the predictive validity of the Dropout Prediction Scale in our sample by evaluating the extent to which a mean score across ages 12, 13, 15, and 17 accurately predicted school dropout by age 19. Participants were considered to have dropped out of school if they did not have a high school diploma and were no longer attending school at age 19 (high school completion is around ages 16 or 17 in Quebec). We used a cutoff of .40 on the Dropout Prediction Scale to classify participants into high versus low dropout risk groups (i.e., 18% versus 82% of the participants), as this cutoff has been shown to maximize specificity and sensitivity in a sample of approximately 40,000 participants (Archambault et al., 2009). We found that, by age 19, 65% of participants considered to be at high risk had dropped out of school (N = 74 out of 113) and 86% of low-risk participants had completed high school (N = 1037 out of 1204), indicating that the Dropout Prediction Scale efficiently classified students at risk of dropout in our sample.

**Computing probabilities for probit coefficients estimated in the random-intercept cross-lagged model**

To convert estimates from paths associated with binary outcomes (i.e., suicidal ideation and attempt) obtained using a probit model to probabilities, we applied a formula developed by Muthen and Muthen (Muthen & Muthen, 2017). The probability of reporting a suicidal behavior (*P*) was calculated using the following formula: *P* (suicidal behavior = 1 | X_1_) = 1 - Φ [(*τ* - *λ*_1_X_1_) / *√θ*], where X_1_ is a given value for the endogenous variable, Φ is the normal distribution function, *τ* is the threshold of the model, *λ* is the regression coefficient associated with the relevant endogenous variable, and *θ* is the residual value of the exogenous variable.

This formula can be extended to compute probabilities for indirect effects involving more than one endogenous variable. For example, the probability of reporting a suicidal behavior at given values of depressive symptoms at ages 12, 13, 15, and 17 can be computed using the following formula: *P* (suicidal behavior = 1 | X_1_, X_2_, X_3_, X_4_) = 1 - Φ [(*τ* - *λ*_1_X_1_ - *λ*_2_X_2_ - *λ*_3_X_3_ - *λ*_4_X_4_) / *√θ*], where X_1_ is a given value and *λ*_1_ is the regression coefficient associated with depressive symptoms at age 12, X_2_ and *λ*_2_, for depressive symptoms at age 13, X_3_ and *λ*_3_, for depressive symptoms at age 15, and X_4_ and *λ*_4_, for depressive symptoms at age 17.

| Table S1. Indirect effects of the random-intercept cross-lagged panel model in the Quebec Longitudinal Study of Child Development (QLSCD)^a^ | | | | |
| --- | --- | --- | --- | --- |
|  |  | **Bootstrapped 95% CI** | |  |
| **Indirect effects** | ***Β*** | **Lower** | **Upper** | ***p*-value** |
| Dep12 -> dep13 -> dep15 -> dep17 -> ideation | .01 | -.002 | .02 | .08 |
| **Dep12 -> dep13 -> dep15 -> dep17 -> attempt** | **.01** | **.0001** | **.02** | **.048** |
| Vic12 -> vic13 -> vic15 -> vic17 -> ideation | .001 | -.002 | .01 | .44 |
| Vic12 -> vic13 -> vic15 -> vic17 -> attempt | .002 | -.001 | .01 | .25 |
| Dep12 -> sch13 -> sch15 -> sch17 -> attempt | .02 | -.09 | .14 | .81 |
| Dep12 -> scho13 -> vic15 -> vic17 -> ideation | -.001 | -.004 | .002 | .39 |
| Dep12 -> scho13 -> vic15 -> vic17 -> attempt | -.002 | -.01 | .002 | .32 |
| Dep 12 -> vic13 -> vic15 -> vic17 -> ideation | .001 | -.001 | .004 | .43 |
| Dep 12 -> vic13 -> vic15 -> vic17 -> attempt | .002 | -.001 | .004 | .28 |
| Abbreviations: CI = confidence intervals; dep = depressive symptoms; sch = school difficulties; vic = peer victimization; 12 = age 12; 13 = age 13; 15 = age 15; 17 = age 17.  ^a^Data were compiled from the final master file of the QLSCD. | | | | |

| Table S2. Descriptive statistics and tetrachoric correlations (with 95% confidence intervals computed using 1,000 bootstraps) between suicide ideation and attempt measures at ages 13, 15, 17, 20 and 23 in the Quebec Longitudinal Study of Child Development (QLSCD)^a^ | | | | | | | | | | | |
| --- | --- | --- | --- | --- | --- | --- | --- | --- | --- | --- | --- |
|  | % (N) | SI 13 | SI 15 | SI 17 | SI 20 | SI 23 | SA 13 | SA 15 | SA 17 | SA 20 | SA 23 |
| SI 13 | .02 (1225) | 1 |  |  |  |  |  |  |  |  |  |
| SI 15 | .03 (1428) | **.46**  **(.11, 63)** | 1 |  |  |  |  |  |  |  |  |
| SI 17 | .04 (1228) | .18  (-.11, .44) | **.49**  **(.23, .64)** | 1 |  |  |  |  |  |  |  |
| SI 20 | .07 (1235) | .16  (-.14, .39) | .28  (-.00, .46) | .22  (-.01, .37) | 1 |  |  |  |  |  |  |
| SI 23 | .07 (1325) | **.49**  **(.27, .63)** | **.29**  **(.02, .46)** | **.30**  **(.04, .46)** | **.46**  **(.30, .58)** | 1 |  |  |  |  |  |
| SA 13 | .02 (1225) | -.04  (-.10, .07) | **.36**  **(.02, .53)** | .37  (-.01, .55) | .29  (-.03, .48) | .21  (-.14, .42) | 1 |  |  |  |  |
| SA 15 | .03 (1428) | **.52**  **(.23, .67)** | -.15  (-.20, -.06) | .19  (-.15, .41) | .07  (-.10, .39) | **.38**  **(.14, .55)** | **.43**  **(.11, .59)** | 1 |  |  |  |
| SA 17 | .02 (1228) | **.42**  **(.05, .62)** | .31  (-.02, .52) | -.15  (-.20, .05) | .19  (-.10, .39) | .29  (-.01, .47) | **.57**  **(.21, .72)** | **.43**  **(.07, .62)** | 1 |  |  |
| SA 20 | .06 (1235) | **.48**  **(.25, .63)** | **.40**  **(.18, .54)** | **.40**  **(.22, .54)** | -.29  (-.35, -.22) | **.22**  **(.03, .37)** | **.49**  **(.22, .66)** | **.53**  **(.35, .66)** | **.59**  **(.42, .70)** | 1 |  |
| SA 23 | .02 (1325) | .07  (-.02, .20) | .12  (-.06, .41) | .25  (-.05, .49) | .10  (-.18, .31) | -.19  (-.24, -.08) | **.41**  **(.09, .65)** | .28  (-.01, .52) | **.35**  **(.05, .58)** | **.56**  **(.33, .70)** | 1 |
| Abbreviations: SI = suicide ideation; SA = suicide attempt.  ^a^Data were compiled from the final master file of the QLSCD. | | | | | | | | | | | |

**Figure S1. Random-intercept cross-lagged panel model predicting suicidal ideation and attempt**

**Figure S2. Cross-lagged panel model predicting suicidal ideation and attempt**

**Figure S3A. Random-intercept cross-lagged panel model including suicide outcomes that account for previous suicide ideation or attempt between ages 13 and 17
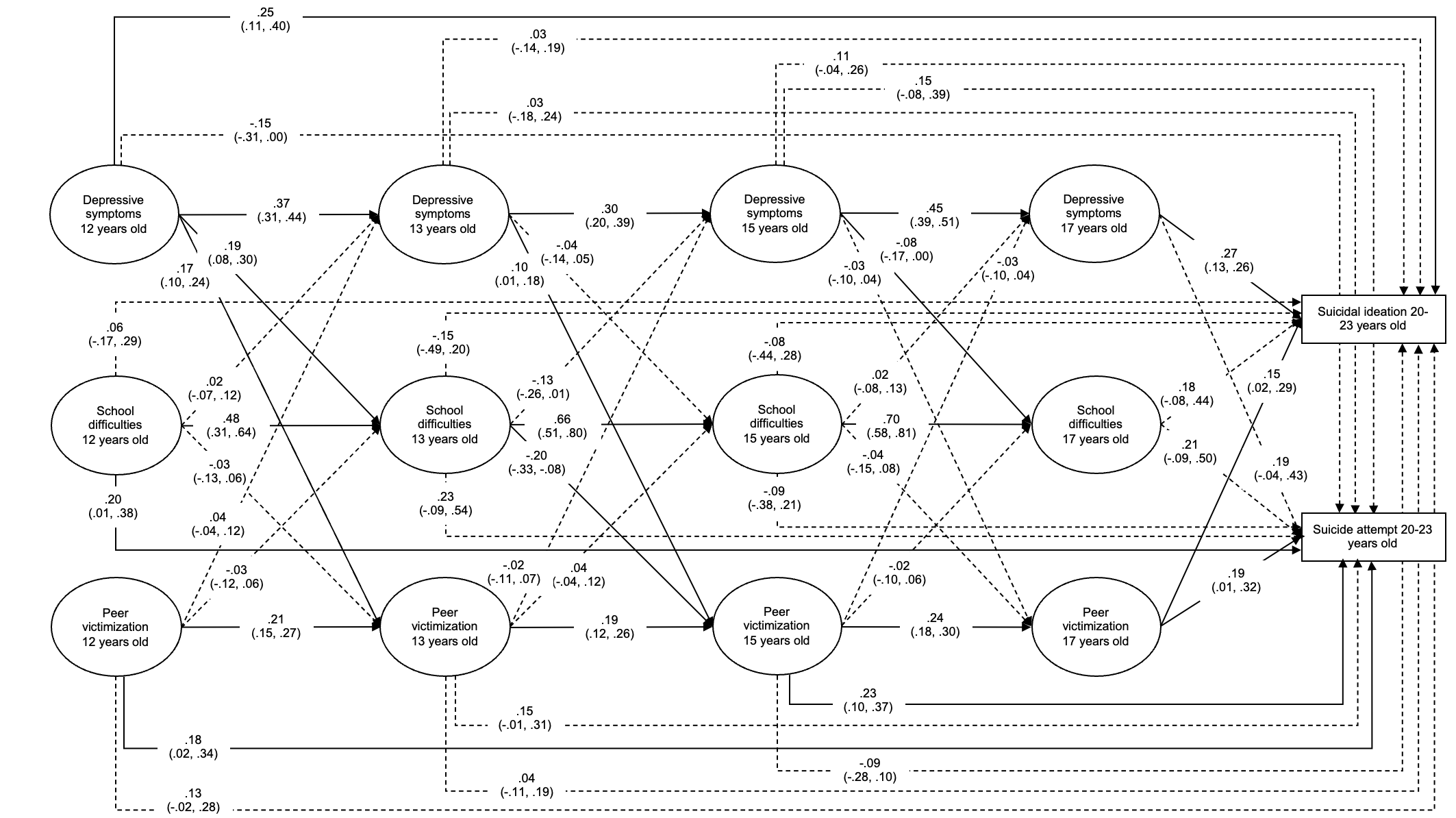
**

**Figure S3B. Random-intercept cross-lagged panel model including suicide outcomes that account for previous suicide ideation or attempt between ages 13 and 17
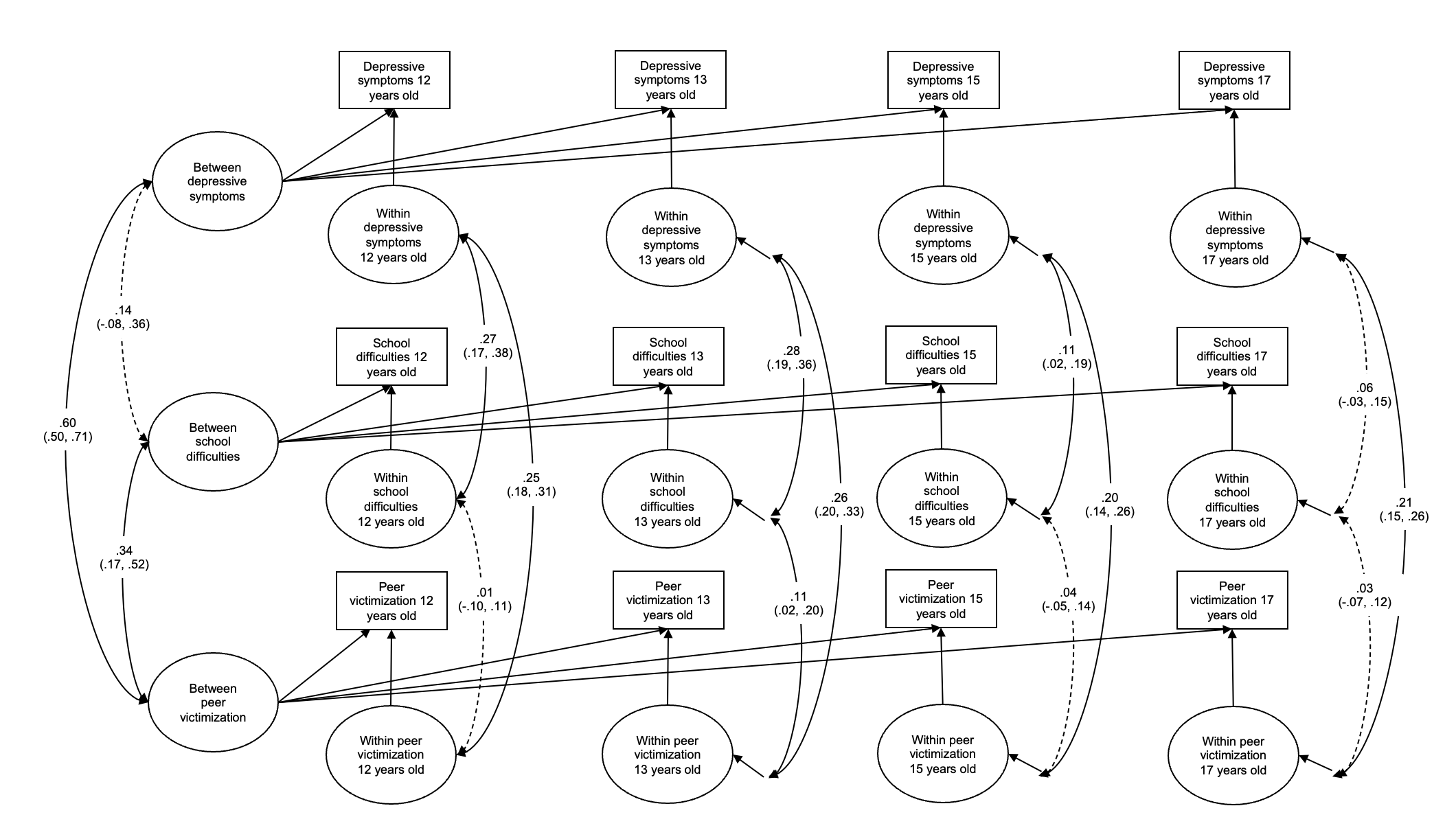
**

*Note.* Outcome variables account for previous suicide ideation and attempt at ages 13, 15, and 17. Suicide ideation or attempt at 20-23 was coded 0 for participants who never reported suicide ideation or attempt at ages 13, 15, 17, 20 and 23 and 1 for participants who reported suicide ideation or attempt for the first time at ages 20 or 23.

**References**

Archambault, I., Janosz, M., Fallu, J. S., & Pagani, L. S. (2009). Student engagement and its relationship with early high school dropout. *Journal of Adolescence*, *32*(3), 651‑670. https://doi.org/10.1016/j.adolescence.2008.06.007

Janosz, M., Le Blanc, M., Boulerice, B., & Tremblay, R. E. (2000). Predicting different types of school dropouts : A typological approach with two longitudinal samples. *Journal of Educational Psychology*, *92*(1), 171‑190. https://doi.org/10.1037/0022-0663.92.1.171

Janosz, M., LeBlanc, M., Boulerice, B., & Tremblay, R. E. (1997). Disentangling the weight of school dropout predictors : A test on two longitudinal samples. *Journal of Youth and Adolescence*, *26*(6), 733‑762. https://doi.org/10.1023/A:1022300826371

Muthen, L. K., & Muthen, B. O. (2017). *Mplus user’s guide*. Muthen & Muthen.
